# Incidence, temporal trends and risk factors of puerperal infection in Mainland China: a meta-analysis of epidemiological studies from recent decade (2010–2020)

**DOI:** 10.1186/s12884-023-06135-x

**Published:** 2023-11-23

**Authors:** Peng Li, Yan Li, Youjian Zhang, Lina Zhao, Xiaohong Li, Junzhe Bao, Jianing Guo, Jun Yan, Ke Zhou, Mingjie Sun

**Affiliations:** 1 Department of Hospital Infection Control, Henan Provincial People’s Hospital, People’s Hospital of Zhengzhou University, Zhengzhou, China; 2 Department of Obstetrics, Henan Provincial People’s Hospital, People’s Hospital of Zhengzhou University, Zhengzhou, China; 3 College of Public Health, Zhengzhou University, Zhengzhou, China; 4 Department of Hospital Infection Control, Henan Province Women and Children’s Hospital, The Third Affiliated Hospital of Zhengzhou University, Zhengzhou, China

**Keywords:** Puerperal infection, Incidence, Risk factors, Meta-analysis, Meta-regression

## Abstract

**Background:**

Puerperal infection (PI) is a severe threat to maternal health. The incidence and risk of PI should be accurately quantified and conveyed for prior decision-making. This study aims to assess the quality of the published literature on the epidemiology of PI, and synthesize them to identify the temporal trends and risk factors of PI occurring in Mainland China.

**Methods:**

This review was registered in PROSPERO (CRD42021267399). Putting a time frame on 2010 to March 2022, we searched Cochrane library, Embase, Google Scholar, MEDLINE, Web of Science, China biology medicine, China national knowledge infrastructure and Chinese medical current contents, and performed a meta-analysis and meta-regression to pool the incidence of PI and the effects of risk factors on PI.

**Results:**

A total of 49 eligible studies with 133,938 participants from 17 provinces were included. The pooled incidence of PI was 4.95% (95%CIs, 4.46–5.43), and there was a statistical association between the incidence of PI following caesarean section and the median year of data collection. Gestational hypertension (OR = 2.14), Gestational diabetes mellitus (OR = 1.82), primipara (OR = 0.81), genital tract inflammation (OR = 2.51), anemia during pregnancy (OR = 2.28), caesarean section (OR = 2.03), episiotomy (OR = 2.64), premature rupture of membrane (OR = 2.54), prolonged labor (OR = 1.32), placenta remnant (OR = 2.59) and postpartum hemorrhage (OR = 2.43) have significant association with PI.

**Conclusions:**

Maternal infection remains a crucial complication during puerperium in Mainland China, which showed a nationwide temporal rising following caesarean section in the past decade. The opportunity to prevent unnecessary PI exists in several simple but necessary measures and it’s urgent for clinicians and policymakers to focus joint efforts on promoting the bundle of evidence-based practices.

## Background

Puerperal infection (PI) is a local or systemic inflammatory reaction caused by the invasion of pathogenic microorganism following childbirth [1]. In severe cases, it could lead to multiple organ failure and even death. As one of the leading threat to maternal health, the global morbidity and mortality of PI in the last two decades were 1.0%-7.2% and 34 per 100,000, respectively [2]. Although the prevalence is declining worldwide, PI still accounts for approximately one tenth of maternal deaths [3], with 94% occurring in low- and middle-income countries (LMICs) [2]. In addition, PI not only seriously affects maternal physical and mental health, but also increases the risk of neonatal infection and causes a non-ignorable proportion of deaths in the first month of their life [4, 5], As we know, the genital tract of normal female is in a state of micro-ecological balance, and its natural immune barrier can defend against most microbial invasion [6]. During pregnancy, childbirth and puerperal period, along with the change of maternal physiological function and internal environment, the self-purification effect of the genital tract decreases and the maternal immunity declines. Especially during childbirth, the genital tract is directly connected with the external environment, which leads to the destruction of the natural defense barrier of the body easily [7]. Besides physiological changes, the improper invasive operations, contamination of medical workers’ hand, equipment and surrounding environment, and unreasonable preventive use of antimicrobial agents in the process of delivery may lead to increased risk of invasion of the endometrium or pelvic connective tissue by anaerobic or gram-negative bacteria [8]. Yet for all that, PI can be well prevented if high risks are recognized and mitigated in a timely manner [9]. And so far, most research on PI has been conducted in high-income countries (HICs), where incidence and risk factors may differ from LMICs due to differences in ethnicity, economics and medical resources. Moreover, the results of some studies on risk analysis of PI are inconsistent or even contradictory. Currently, a comprehensive and contemporary assessment and update of the epidemiology and risk of PI in LMICs is critical but absent. Therefore, through literature retrieval and evaluation, data extraction and analysis, this study attempts to identify the temporal trends and risk factors of PI occurring in Mainland China. This will help provide an evidence base for stewardship initiatives and further research on PI prevention.

## Materials and methods

### Search strategy

Putting a time frame on 2010 to the present, we searched the databases (sort them alphabetically) of Cochrane library, Embase, Google Scholar, MEDLINE and Web of Science for relevant English language literatures, and the databases of China biology medicine (CBM), China national knowledge infrastructure (CNKI) and Chinese medical current contents (CMCC) for relevant Chinese language literatures. Search strategies were customized to each electronic database and based on the following combinations of key search terms: maternal, obstetric, puerperal, postpartum, puerperium, after birth, after delivery, infection, sepsis, endometritis, risk factors etc. In case of duplicate publications, we included only the study with most detailed information. Moreover, the references cited in the identified literatures were further searched by manual retrieval.

### Selection criteria

All observational, longitudinal studies (case-controls, prospective or retrospective cohorts) were included in our review if they recruited participants who had undergone childbirth in Chinese hospitals, reported on outcomes of PI following delivery and/or reported on the associations of PI with any maternal-related factors (sociodemographic and clinical characteristics, such as family economic condition, body mass index, comorbidities, and past medical history), delivery-related factors (e.g., delivery mode, duration of labor, soft birth canal injuries or not), or hospital-related factors (e.g., rationality of prophylactic antibiotics using, the scale or level of hospital). Besides, for the studies on risk factors associated with PI, the following criteria should be met: (1) The definitions of exposure were clear and basically consistent; (2) The OR value with 95% confidence intervals (CIs) could be calculated or converted from the raw data provided. And studies were excluded if they: (1) Fewer than 20 participants; (2) Only a subgroup of puerperae with higher risk (e.g., hypertensive disorder, diabetes mellitus, older childbearing age) of PI other than the general population of puerperae; (3) Duplicate publications with identical participants; (4) Case report, review, poster or conference abstracts; (5) Incomplete or inconsistent raw data.

### Outcome and exposure definitions

PI was defined as maternal in-hospital infection after childbirth and post-discharge infection occurring at any time during the 6-week postpartum period, which consisted of genital tract infection, urinary tract infection, wound infection following cesarean section, infectious peritonitis, sepsis or other specified infectious complications of surrounding tissues [9]. And we categorized the PIs in the included studies according to the code O85 (puerperal sepsis) and O86 (Other puerperal infections) in the International Classification of Diseases, 10th revision (ICD-10, Version: 2016) [10]. Furthermore, to avoid misclassification caused by unclear definitions of exposure in various studies, some exposure factors were defined as follows: (1) Elderly puerpera. Women aged 35 and older at the time of delivery; (2) Gestational hypertension. First appearance of hypertension during pregnancy with systolic blood pressure ≥ 140 mmHg and/or diastolic blood pressure ≥ 90 mmHg, which referred to the guidelines by the American College of Obstetricians and Gynecologists (ACOG) and the Obstetrics and Gynecology Branch of Chinese Medical Association (OGBCMA) [11, 12]; (3) Gestational diabetes mellitus (GDM). Diabetes mellitus occurred during pregnancy, while glucose metabolism was normal before pregnancy. And fasting blood glucose and/or oral glucose tolerance test results met the diagnostic criteria in the guidelines by the American Diabetes Association and ACOG [13, 14]; (4) Anemia during pregnancy (AP). Hemoglobin concentration < 110 g/L during pregnancy according to the recommendations of WHO and the Chinese Society of Perinatal Medicine [15, 16]; (5) Premature delivery. Delivery occurred between 28 and 37 gestational weeks; (6) Operative vaginal delivery. A procedure in which the fetal head is directly drawn by forceps or aspirators during the second stage of labor to accelerate or achieve vaginal delivery [17]; (7) Prolonged labor. For the first stage of labor, the incubation period > 20 h for primipara and > 14 h for multipara. For the second stage of labor, if epidural block was performed, the progression-free period > 4 h for primipara and > 3 h for multipara; if there was no epidural block, the progression-free period > 3 h for primipara and > 2 h for multipara [18, 19]; (8) Premature rupture of membrane (PROM). A sudden vaginal discharge or uncontrolled leakage of urine appeared before delivery, and vaginal examination showed that amniotic fluid mixed with fetal fat flowed out of the cervix, and/or the smear test and biochemical test referred to ACOG guidelines [20]; (9) Postpartum hemorrhage. The bleeding volume ≥ 500 mL for vaginal delivery and ≥ 1 000 mL for cesarean delivery within 24 h after delivery [21, 22].

### Data extraction and quality assessment

Two authors (L.P. and L.Y.) independently extracted data, which included first author, research method, study location, year of data collection, hospital level, sample size, social characteristics of participants, exposure factors, number of exposed and no exposed participants, number and subtype of PIs. And we contacted the authors to provide the necessary data. This review was registered on the PROSPERO database with a registration number of CRD42021267399 and conducted according to PRISMA guidelines [23]. The quality of studies and risk of bias were quantitatively assessed by using the corresponding critical appraisal tools from Joanna Briggs Institute (JBI) for studies reporting incidence/prevalence data [24], case-control studies and cohort studies [25], which include nine, ten and twelve “stars” respectively. Two authors (L.P. and B.J.Z.) independently scored each study and any disagreement between authors was resolved by consensus.

### Statistical analysis

Given the incidence of PI is close to zero in most studies, the individual study-specific incidences were presented via the score method to avoid yielding inadmissible values (i.e. less than zero) [26]. The subgroup and overall incidences with 95% Wald CIs were pooled by using the random-effects inverse-variance model and Freeman-Tukey double arcsine transformation and back transformation to approximate a normal distribution and stabilize the variance [27]. And the temporal trends in incidence were evaluated using the median year of data collection reported by included studies, as previously reported [28].

The measures of association between exposures and PI were presented as OR with 95% Wald CIs. The *x*^2^ test were used to calculate the Cochran’s Q statistic, along with I^2^ to quantify the between-study heterogeneity, according to which the random- or fixed-effects model was chosen to pool the subgroup and overall OR values with log transformation and back transformation. For studies of specific risk factors with heterogeneity, the random effects meta-regression was performed to identify the sources of heterogeneity, and then a subgroup analysis was conducted to assess the effects of these sources on heterogeneity. Next, to detect publication bias quantitatively, the fail-safe N coefficient was adopted to calculate the potential number of studies that would have been needed to reverse the current effect [29], and the Harbord weighted linear regression method was performed to measure the association between the sample size and pooled OR value [30], as the number of included studies on specific risk factors is generally less than 20 [31]. Considering the low power of Cochran’s Q Test and meta-regression and the poor quality of partial included studies, we set the significance level (α) to 0.10 to reduce the probability of false negatives and to identify as many factors contributing to heterogeneity as possible. All statistical analyses were conducted using STATA SE 15.1 (Stata Corporation, Texas, USA).

## Results

### Study selection and characteristics

**Fig. 1:**
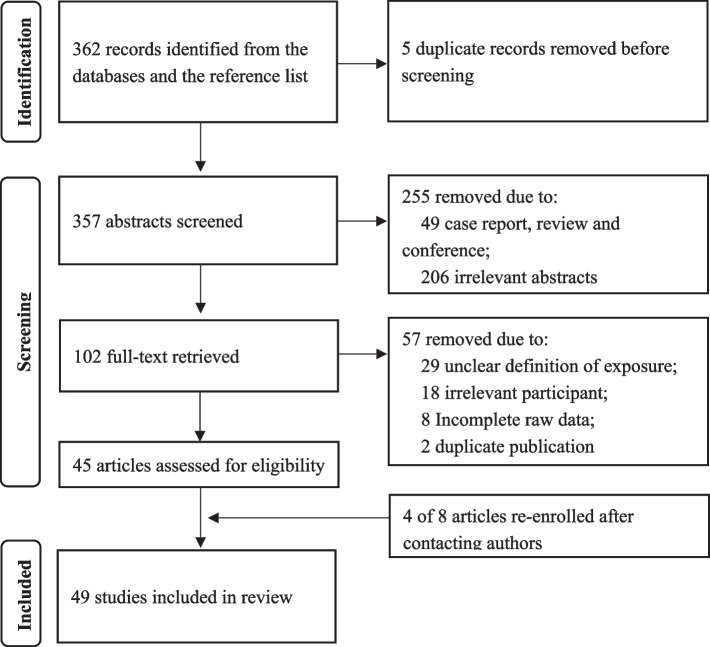
Flow diagram of study selection

A total of 49 eligible studies with 133,938 participants from 17 provinces in Mainland China were included after retrieval and screening (Fig. 1) [32–80]. Five studies were case–control studies, five were prospective cohort studies, and the remainder were retrospective cohort studies. Of these, 46 studies published from 2004 to 2021 reported the incidence of PI, 30 (65.21%) of which had a JBI score greater than 5, with no significant temporal trend in JBI scores (Pearson *r* = 0.208, *P* = 0.165). And 27 studies published from 2006 to 2021 reported the risk factors for PI, 25 (92.59%) of which had a JBI score greater than 6, with a upward temporal trend in JBI scores (Pearson *r* = 0.454, *P* = 0.017). The detailed information and quality of included studies are available in Table 1.

**Table 1:** Profile of the 49 eligible studies

Number	Author(survey time), province	Study design	Hospital level	Number of PI/ total	JBI score for incidence	JBI score for risk factors
1	Yuan Q (201311–201610), Jiangsu	RC	tertiary	298/37267	8	-
2	Zhang DH (201105–201306), Guangdong	PC	secondary	10/310	5	-
3	Zeng WQ (201701–201812), Jiangxi	RC	secondary	44/1972	6	-
4	Wu J (201102–201503), Jiangsu	RC	tertiary	86/1927	8	-
5	Liu YR (201504–201703), Sichuan	PC	secondary	12/51	4	-
6	Xie QB (2018), Hunan	RC	tertiary	200/20000	6	-
7	Zeng XM (200901–201003), Jiangxi	RC	secondary	6/2008	5	-
8	Chang R (201501–201612), Shaanxi	RC	tertiary	37/2846	9	-
9	Zhang QL (201407–201412), Shandong	PC	tertiary	32/369	6	-
10	Xun SL (201501–201503), Jiangsu	RC	tertiary	195/1030	6	-
11	Liu JF (201501–201601), Neimenggu	RC	secondary	9/1000	3	-
12	Wu XM (201701–201901), Guangdong	RC	secondary	22/1000	4	-
13	Huang ZH (201604–201609), Guangdong	RC	tertiary	44/580	7	-
14	Zhang LJ (2013–2017), Neimenggu	RC	tertiary	39/240	3	-
15	Wu P (201401–201410), Shandong	RC	tertiary	12/320	5	-
16	Weng KN (2014–2016), Zhejiang	RC	secondary	42/899	8	-
17	Zhang ZT (201509–201609), Guizhou	RC	tertiary	32/120	3	-
18	Luo YJ (201703–201705), Guangdong	RC	secondary	52/1641	5	-
19	Qiu H (2008–2011), Shandong	RC	secondary	56/568	4	-
20	Yi WN (201701–201901), Shandong	PC	secondary	9/100	4	-
21	Wen Z (201802–202008), Guangxi	PC	tertiary	3/100	3	-
22	Luo QZ (201301–201604), Zhejiang	RC	secondary	24/280	4	-
23	Yue LY (200601–201111), Shanxi	RC	tertiary	34/1496	6	8
24	Wang CY (2017–2019), Shaanxi	RC	tertiary	31/190	8	9
25	He LP (201601–201701), Shanxi	RC	tertiary	64/800	5	9
26	Huang XA (201402–201603), Zhejiang	RC	tertiary	80/3000	7	10
27	Huang SJ (201506–201610), Hainan	RC	tertiary	17/483	7	9
28	Ai D (2015–2019), Sichuan	RC	tertiary	89/1957	8	9
29	Shi SY (201612–201807), Liaoning	RC	secondary	82/1000	8	7
30	Xiong F (2012–2015), Sichuan	RC	tertiary	78/1263	7	9
31	Zhang R (2017), Zhejiang	RC	tertiary	187/24195	8	10
32	Fan LY (201001–201410), Zhejiang	RC	secondary	85/1828	6	8
33	Fang J (201702–202001), Zhejiang	RC	tertiary	92/2138	9	11
34	Zhang LY (200501–201101), Guangxi	RC	tertiary	120/5000	5	7
35	Li X (201710–201909), Henan	RC	tertiary	219/1560	9	11
36	Zhao SQ (201402–201512), Ningxia	CC	secondary	98/300	6	7
37	Zhang J (201601–201810), Henan	RC	tertiary	122/2800	7	10
38	Lin L (201807–201912), Liaoning	RC	tertiary	34/500	8	10
39	Liu WL (2013–2015), Henan	RC	tertiary	64/1026	9	11
40	Wang M (201008–201208), Shandong	RC	secondary	27/400	5	7
41	Huang SL (201403–201602), Guangdong	RC	tertiary	20/403	8	9
42	Qu ZY (201607–201912), Liaoning	RC	tertiary	47/1123	9	11
43	Shen YL (2014–2015), Zhejiang	CC	secondary	80/4653	8	8
44	He LJ (201001–201308), Zhejiang	RC	tertiary	53/826	7	7
45	Chen YY (201408–201810), Henan	RC	tertiary	91/1521	8	10
46	Liao D (201701–201910), Hainan	RC	tertiary	20/400	9	11
47	Li QX (2014), Tianjin	CC	secondary	40/80	-	7
48	Lan FP (201601–201710), Guangxi	CC	secondary	68/136	-	3
49	Cai LF (201101–201501) Zhejiang	CC	secondary	72/232	-	3

### Incidence of PI

**Fig. 2:**
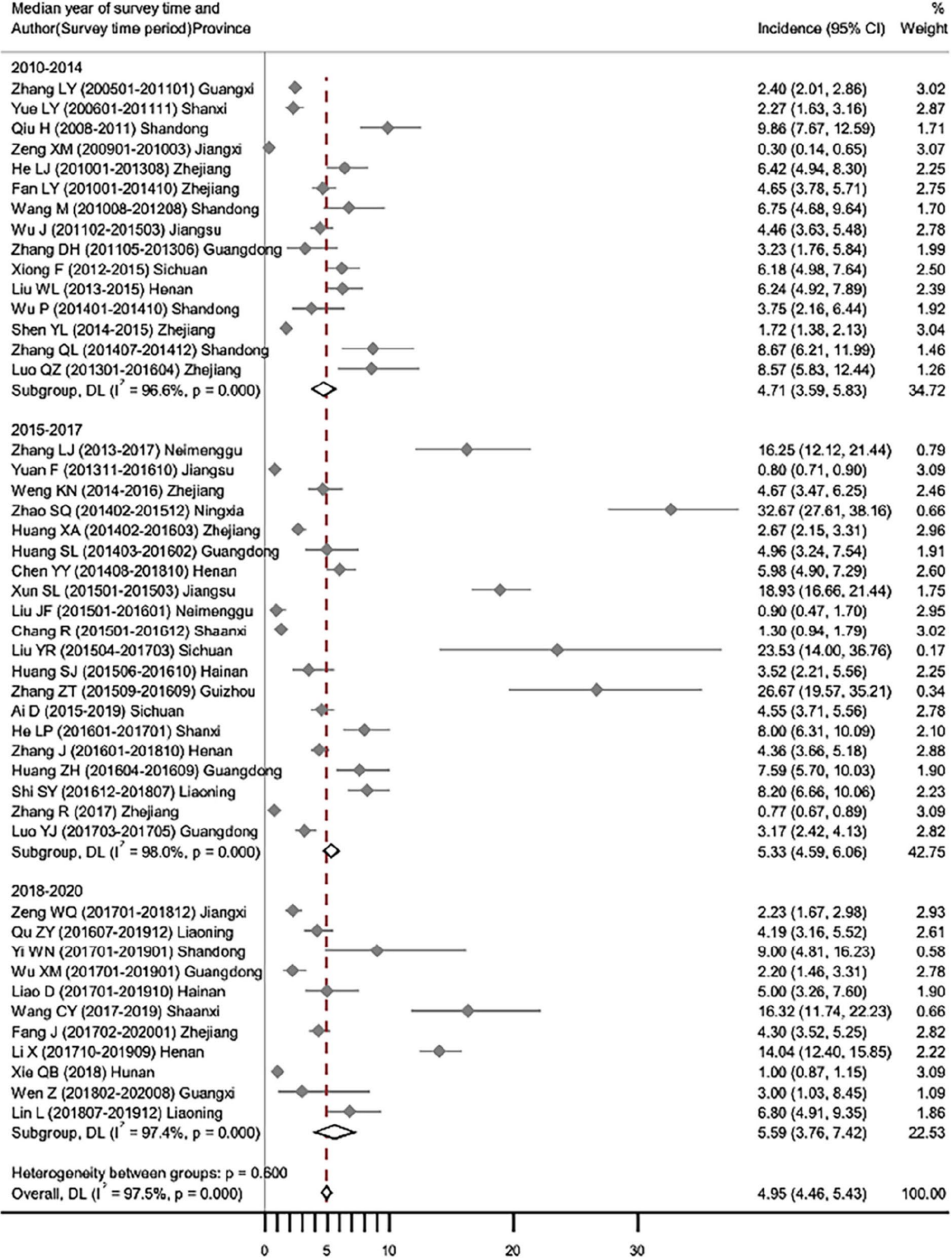
Forest plot for studies reporting the incidence of PI, grouped and ordered by the median year of data collection

**Fig. 3:**
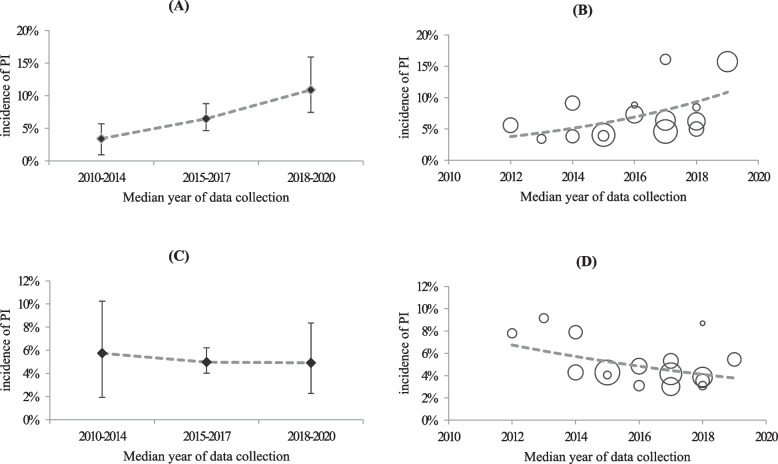
Temporal trends in incidence of PI stratified by delivery modes after excluding studies with JBI score of 6 or less. **a** Incidence of PI following cesarean section (with 95% CI); **b** Meta-regression bubble plot for incidence of PI following cesarean section; **c** Incidence of PI following vaginal delivery (with 95% CI); **d** Meta-regression bubble plot for incidence of PI following vaginal delivery

The incidence of PI ranged from 0.30% to 32.67% over the study period, with a random-effects estimation of pooled incidence of 4.95% (95%CIs, 4.46–5.43). The forest plot for studies reporting the incidence of PI are available in Fig. 2. In meta-regression analysis, incidence of PI were not statistically associated with sample size, hospital level or the median year of data collection, but with study quality (*P* = 0.176, 0.783, 0.545 and 0.009, respectively) (Table 2). After excluding studies with JBI score of 6 or less, the pooled incidence decreased to 4.58% (95%CIs, 3.45–5.86), while still remained non-significant association with the median year of data collection (*P* = 0.491). After stratified by delivery mode, the incidence of PI was 7.11% (95%CIs, 4.91–9.92) following caesarean section and 4.75% (95%CIs, 2.74–6.89) following vaginal delivery. And there was a significant association between incidence of PI following caesarean section and the median year of data collection (*P* < 0.01), but not between incidence of PI following vaginal delivery and the median year of data collection (*P* = 0.920) (Fig. 3).

**Table 2:** Effects of study characteristics on the pooled incidence of PI

Study characteristics	Incidence (95% CIs)	Meta-regression	
		b coefficient	*P* value
*Sample size*			
≤ 1000	5.38% (4.29%, 6.47%)	-0.013	0.176
> 1000	4.51% (4.14%, 4.88%)		
*Hospital levels*			
Secondary hospital	4.68% (3.37%, 5.96%)	-0.006	0.783
Tertiary hospital	4.97% (3.09%, 7.35%)		
*Median year of data collection*			
2010–2014	4.71% (3.59%, 5.83%)	0.009	0.545
2015–2017	5.33% (4.59%, 6.06%)		
2018–2020	5.59% (3.76%, 7.42%)		
*JBI scores*			
1–4	8.94% (4.29%, 15.24%)	-0.021	0.009
5–6	5.33% (3.17%, 8.00%)		
≥ 7	4.58% (3.45%, 5.86%)

### Risk factors of PI

#### The overall OR values

The factors of 1) gestational hypertension, GDM, multipara, genital tract inflammation and AP before childbirth, 2) caesarean section, episiotomy, PROM and prolonged labor during childbirth, 3) placenta remnant and hemorrhage after childbirth were significantly associated with PI (*P* < 0.01) (Table 3).

**Table 3:** Results of the pooled OR, heterogeneity and publication bias detection

Risk factors	Heterogeneity test	OR (95% CI)	Number of included studies	Fail-safe number^c^	Harbord’s modified test	
					Intercept	*P* value
*Before childbirth*						
Elder	*P* = 0.79, I^2^ = 0%^a^	1.20 (0.99, 1.45)^b^	7	-	-	-
Gestational hypertension	*P* < 0.01, I^2^ = 90%	2.14 (1.77, 2.58)	8	399	6.67	< 0.01
GDM	*P* < 0.01, I^2^ = 84%	1.82 (1.52, 2.17)	8	253	4.96	< 0.01
Primipara	*P* = 0.62, I^2^ = 0%^a^	0.81 (0.67, 0.97)	6	4	2.26	0.07
Multiple pregnancy	*P* = 0.12, I^2^ = 48%^a^	1.44 (0.91, 2.25)^b^	4	-	-	-
Genital tract inflammation	*P* = 0.15, I^2^ = 43%^a^	2.51 (2.09, 3.02)	9	275	-2.68	0.14
AP	*P* = 0.14, I^2^ = 43%^a^	2.28 (2.01, 2.59)	15	808	0.49	0.63
*During childbirth*						
Premature delivery	*P* = 0.20, I^2^ = 32%^a^	1.21 (0.93, 1.57)^b^	6	-	-	-
Caesarean section	*P* < 0.01, I^2^ = 77%	2.03 (1.80, 2.29)	19	580	0.79	0.61
Operative vaginal delivery	*P* = 0.15, I^2^ = 44%^a^	1.23 (0.94, 1.62)^b^	4	-	-	-
Episiotomy	*P* = 0.41, I^2^ = 0%^a^	2.64 (1.76, 3.94)	4	35	2.19	0.09
PROM	*P* < 0.01, I^2^ = 86%	2.54 (2.23, 2.89)	19	1165	5.31	0.01
Prolonged labor	*P* = 0.12, I^2^ = 48%^a^	1.32 (1.10, 1.57)	8	41	-2.00	0.44
*After childbirth*						
Placenta remnant	*P* = 0.25, I^2^ = 23%^a^	2.59 (1.94, 3.48)	7	118	2.05	0.23
Postpartum hemorrhage	*P* = 0.65, I^2^ = 0%^a^	2.43 (2.13, 2.77)	18	1151	1.30	0.05

^a^No significant heterogeneity existed among included studies (*P* > 0.10 and I^2^ < 50%)

^b^The pooled OR value has no statistical significance (*P* > 0.01)

^c^The number of studies required to reverse the effects are calculated on the condition of *P* = 0.05

#### Subgroup analysis

There were significant heterogeneities among the included studies on the factors of gestational hypertension, GDM, caesarean section and PROM (*P* < 0.10 and I^2^ > 50%) (Table 3). Meta-regression analysis for these factors showed that the pooled OR were statistically associated with sample size for gestational hypertension and GDM (*P* = 0.07 and 0.08), hospital levels for caesarean section and PROM (*P* = 0.01 and < 0.01), study design for caesarean section and PROM (*P* = 0.03 and 0.01), JBI scores for PROM (*P* = 0.06) (Table 4). After stratified by these study characteristics, the heterogeneity still existed but decreased among the included studies on the factors of GDM, caesarean section and PROM, while disappeared among the included studies on the factors of gestational hypertension (Table 5).

**Table 4:** The source of heterogeneity among included studies identified by meta- regression

Study characteristics^a^	Gestational hypertension		GDM		Caesarean section		PROM	
	b coefficient	*P* value	b coefficient	*P* value	b coefficient	*P* value	b coefficient	*P* value
Sample size	-0.91	0.07^b^	-0.88	0.08^b^	-0.36	0.22	-0.44	0.26
Hospital levels	-0.81	0.15	-0.60	0.31	-0.76	0.01^b^	-1.17	< 0.01^b^
Median year of data collection	-0.73	0.28	0.26	0.67	0.10	0.69	0.01	0.97
Study design	-0.48	0.46	-0.53	0.42	0.71	0.03^b^	-1.13	0.01^b^
JBI scores	-0.32	0.37	-0.29	0.42	-0.21	0.34	-0.52	0.06^b^

^a^The grouping of JBI scores was consistent with that in Table 5

^b^The study characteristic was the source of heterogeneity among included studies on the corresponding risk factors of PI (*P* < 0.10)

**Table 5:** Effects of study characteristics on the pooled OR values by subgroup analysis

Risk factors	Study characteristics				Heterogeneity test	OR (95% CI)	Number of included studies
	Sample size	Hospital levels	Study design	JBI scores			
Gestational hypertension	≤ 1000	-	-	-	*P* = 0.25, I^2^ = 27%^a^	5.57 (3.90, 7.94)	4
	> 1000	-	-	-	*P* = 0.63, I^2^ = 0%^a^	1.53 (1.22, 1.91)	4
GDM	≤ 1000	-	-	-	*P* = 0.14, I^2^ = 45%^a^	4.17 (2.87, 6.08)	4
	> 1000	-	-	-	*P* = 0.01, I^2^ = 62%	1.43 (1.16, 1.77)	4
Caesarean section	-	Secondary	-	-	*P* = 0.03, I^2^ = 58%	3.63 (2.85, 4.64)	6
	-	Tertiary	-	-	*P* = 0.71, I^2^ = 0%^a^	1.69 (1.47, 1.94)	13
	-	-	CC	-	*P* = 0.02, I^2^ = 66%	3.72 (1.16, 4.96)	5
	-	-	RC	-	*P* = 0.20, I^2^ = 31%^a^	1.78 (1.56, 2.03)	14
PROM	-	Secondary	-	-	*P* = 0.18, I^2^ = 35%^a^	8.48 (6.21, 11.56)	6
	-	Tertiary	-	-	*P* < 0.01, I^2^ = 61%	1.82 (1.57, 2.12)	13
	-	-	CC	-	*P* = 0.13, I^2^ = 46%^a^	8.99 (6.27, 12.90)	5
	-	-	RC	-	*P* = 0.03, I^2^ = 57%	1.94 (1.68, 2.24)	14
	-	-	-	1–4	*P* = 0.75, I^2^ = 0%^a^	5.02 (2.79, 9.03)	2
	-	-	-	5–7	*P* < 0.01, I^2^ = 83%	6.19 (4.77, 8.04)	5
	-	-	-	≥ 8	*P* = 0.15, I^2^ = 45%^a^	1.78 (1.52, 2.09)	12

^a^No significant heterogeneity exists among included studies (*P* > 0.10 and I^2^ < 50%)

#### Sensitivity analysis

For the factors with fewer than 7 included studies, the stability of their pooled OR values was assessed by removing one study at a time. The results showed little change in the pooled OR values after each study was removed, except that the pooled OR value for the factor of multiple pregnancy turned to be statistically significant when the study with reference No.62 was removed (*P* < 0.01) (Table 6).

**Table 6:** Results of sensitivity analysis by removing one study at a time

Study removed	OR (95% CI)				
	Primipara	Multiple pregnancy	Premature delivery	Operative vaginal delivery	Episiotomy
54	-	-	-	-	2.65 (1.71, 4.10)
55	-	-	-	-	3.88 (2.09, 7.22)
56	0.81 (0.67, 0.97)	-	-	1.43 (0.94, 1.94)	2.55 (1.64, 3.96)
62	-	2.02 (1.14, 3.59)^a^	1.23 (0.92, 1.64)	-	-
64	0.78 (0.67, 0.96)	1.13 (0.63, 2.03)	-	1.24 (0.89, 1.73)	-
66	0.87 (0.69, 0.98)	-	1.34 (0.94, 1.91)	-	-
68	0.80 (0.65, 0.98)	-	1.08 (0.81, 1.43)	-	-
69	0.79 (0.65, 0.96)	1.27 (0.77, 2.09)	1.22 (0.93, 1.59)	-	-
70	0.77 (0.63, 0.95)	1.55 (0.97, 2.47)	1.21 (0.92, 1.59)	-	-
74	-	-	-	1.24 (0.94, 1.65)	2.28 (1.65, 3.50)
76	-	-	-	1.02 (0.73, 1.43)	-
77	-	-	1.24 (0.95, 1.62)	-	-

^a^After removing the study with reference No.62, the heterogeneity among included studies on the factor of multiple pregnancy decreased (*P* = 0.31, I^2^ = 14%)

### Publication bias

The publication bias was detected for the factors statistically associated with PI (Table 3). If there had been publication bias, at least 399, 253, 4, 275, 808, 580, 35, 1165, 41, 118 and 1151 studies, respectively, would have been required to reverse the current effects of gestational hypertension, GDM, primipara, genital tract inflammation, AP, caesarean section, episiotomy, PROM, prolonged labor, placenta remnant and Postpartum hemorrhage (*P* = 0.05). The result of Harbord weighted linear regression test showed that asymmetry with larger intercept value existed in the funnel plots of the factors of gestational hypertension and GDM, and there were significant correlations between the sample size and OR values of these two factors’ included studies (*P* < 0.01).

## Discussion

### Major findings

To our knowledge, this is the first systematic review on synthesizing and evaluating the incidence, temporal trends and risk factors of PI in Mainland China. The incidence of PI ranged widely from 0.30% to 32.67% across individual studies and averaged approximately 4.95% in pooled analysis. The incidence of PI following caesarean section was higher than that of vaginal delivery and appeared to be a temporal rising over the past decade.

Various maternal- and hospital-related factors before, during and after childbirth showed their associations with PI. And our analysis found no statistically significant association between age, multiple pregnancy, premature delivery or operative vaginal delivery and PI, whereas primipara was associated with decreased risk of PI.

### Comparison and explanations

Understanding the epidemiology of PI is the first step towards evidence-based clinical practice. And in this analysis, we found a much higher incidence of PI than in a recent study covered 46 countries between 2005 and 2016, with 90% of the data coming from HICs [81]. Even so, the incidence of PI in mainland China may be slightly underestimated. Firstly, the data from all included studies did not cover home deliveries, which accounts for a tiny fraction of deliveries under China’s current maternity insurance system and household registration policy. Secondly, loss to follow-up after discharge or mild symptoms without readmission may cause the diagnosis to be missed [82]. Finally, low current rate of pathogenic detection before infection diagnosis or antibiotic prescription is a common problem in Chinese hospitals [83], and nonspecific signs and symptoms may cause the diagnosis of infection to be not reported or at least delayed due to a lack of clear etiological evidence.

Although the general incidence of PI has not changed across the country, it should not be ignored that our results also showed the incidence of PI following caesarean section was higher than that of vaginal delivery and has increased strongly in the past decade. As we know, with the continuous development of perinatal medicine and the improvement of surgical safety, the rate of cesarean section is increasing globally [2]. Due to the misunderstanding of cesarean section among puerperae and some medical personnel, economic incentives for hospitals and relaxing of population policy, the annual rate of cesarean section in China ascended from 28.8% in 2008 to 36.7% in 2018, ranking first in Asia [84]. Nevertheless, as an unnatural and traumatic method of delivery, cesarean section has short- and long-term effects on both mothers and newborns and is recognized as an independent and high risk factor for PI [85]. The pooled OR value of cesarean section we calculated was higher than that of a study on meta-analysis of the association between PI and cesarean section without medical indication from 28 countries [86], and lower than the result from a 5-year cohort study covered 32,468 women in Denmark [87], which could reflect regional differences in perioperative management and infection control practices [88]. As our findings showed, there existed an upward tendency of PI following cesarean section in recent 10 years, which further validates the importance and urgency of implementing effective bundle of cesarean section management, such as regulating surgical indications, practicing infection prevention measures, improving the service capacity of midwifery, providing better delivery analgesia services and so on.

In addition to caesarean section, episiotomy is another invasive operation characterized by high rate and high risk of PI as our findings indicated. As an internationally controversial operation in the past 10 years, episiotomy could alleviate the pain of prolonged labor, accelerate the delivery of fetus, prevent the aggravation of fetal distress, and even reduce risks of several maternal complications, which were thought to include subsequent urinary and fecal incontinence, pelvic floor dysfunction and prolapse, and sexual dysfunction [89]. However, in view of the potential intraoperative contamination, perineal trauma and psychological impact on puerperae [90], a growing body of research suggests that episiotomy not only increases the chance of 3rd and 4th degree lacerations of the perineum and [91], as shown in our study, also increases the risk of PI. Although obstetric textbooks for learning and teaching in Chinese medical colleges have stated that episiotomy is not performed routinely and the OGBCMA officially clarified its surgical indications in 2016 [92], the episiotomy rate in China is still much higher than that recommended by WHO, and more than half of episiotomies in primipara and one-fourth in multipara have no indication [93]. In order to reduce unnecessary episiotomy, midwives should be fully trained and follow the indications recommended in the guidelines, while regulatory bodies of governments and hospitals should strengthen their supervision by incorporating the implementation rate and correct rate of episiotomy into technical assessment and performance evaluation.

Moreover, in common with other studies, we found the underlying diseases such as gestational hypertension and GDM, prenatal symptoms of genital tract inflammation and AP, as well as placenta remnant and hemorrhage after childbirth, would increase the probability of PI to varying degrees. The mainstream explanation is that the existence of above risk factors leads to the hemodynamic disorder of endometrium easily, and hyperosmosis and hyperglycemia increase the colonization rate of pathogens, thus promoting the occurrence of infection. In addition, PROM changes the closed environment of the uterine cavity and weakens both the bactericidal effect of lysozyme in amniotic fluid and the self-purification effect of vagina, which leads to the imbalance of bacterial flora in maternal genital tract and increases the chance of intrauterine infection [94]. Therefore, standardized monitoring and timely correction of the underlying diseases and prenatal symptoms before childbirth should be routinely and effectively conducted. In addition to the necessary and careful examination of the integrity of the placenta and membranes after childbirth, every obstetric medical staff should have the emergency response capability to identify and properly deal with postpartum hemorrhage, and gradually improve the level of diagnosis and treatment of it through continuous learning and training. More importantly, obstetric departments should establish PI prediction models or scoring tools suitable for their own hospitals according to these maternal-related risk factors of PI, so as to quantitatively and timely identify puerperae at high risk of PI and to take targeted early intervention measures.

What is also worth noticing is that, although there has been no clear consensus on the effect of parity on PI until now, our results showed that primiparae were less likely to develop PI than multiparae. One potential explanation is that the vaginal microbial environment is affected and modified strongly by pregnancy history [95]. The absence of previous deliveries is a major predictor of Lactobacillus spp., which plays an important role in stabilizing the vaginal microbiota and protects the upper genital tract from colonization and infection by pathogens [96], while the relative abundance of Lactobacillus spp. is lower among multiparae [97]. Therefore, compared with the routine health education and care for primiparae who have no childbirth experience, perinatal monitoring and postpartum care for multipara should not be taken lightly.

### Limitations

The findings of our incidence rate-related analysis require interpretation with caution, given the following limitations: (i) We adopted the WHO definition of PI, but acknowledged that very few surveys were able to investigate infection up to 6 weeks postpartum, and some studies only interviewed or assessed puerperae during hospitalization; (ii) The heterogeneity was observed among included studies when pooling the incidence. In addition to the differences in study quality identified by meta regression, sources of heterogeneity may also be attributed to a variety of other parameters with limited access, such as regional differences in economic levels and population characteristics, hospital differences in diagnostic procedures, perioperative managements and infection control practices; (iii) The analysis of temporal trends in PI rates was based on the median year of study data collection, which may have resulted in an inability to accurately capture the specific perinatal and follow-up periods. Meanwhile, there are two major limitations to our risk analysis: (i) The studies included were observational in design, which may be subject to selection, recalling or attrition bias, hence the confidence of effect estimates may be biased and the validity of the findings could have been limited; (ii) Due to lack of data, our analysis could not identify the contribution of other potential risk factors, such as maternal financial situation and psychological characteristic, nor could it explore and reduce inter-study heterogeneity by stratification through a number of other important confounding factors, such as hospital location and PI type.

## Conclusions

Our study details the changing epidemiology and various risk of PI. Maternal infection is revealed to be a still common and crucial complication during puerperium in Mainland China, and it has shown a strong nationwide temporal rising following caesarean section in the past decade. Risk factors for PI include 1) gestational hypertension, GDM, multipara, genital tract inflammation and AP before childbirth, 2) caesarean section, episiotomy, PROM and prolonged labor during childbirth, 3) placenta remnant and hemorrhage after childbirth. The noticeable effects of various maternal- and hospital-related factors on the risk of PI indicates that the opportunities for preventing PI exist in several simple but necessary measures, such as strengthening prenatal screening and treatment of underlying diseases, regulating surgical indications, promoting restrictive over routine episiotomy, standardizing aseptic operation during labor, enhancing postpartum care and monitoring of uterine contraction. Moreover, great attempts are warranted to reduce unnecessary PI through evidence-based education, training and management. In an era of declining global fertility but rising antimicrobial resistance, our findings highlight the importance and urgency for clinicians and policymakers to focus joint efforts on promoting the bundle of evidence-based practices to prevent PI.

## Data Availability

The datasets used and/or analysed during the current study are available from the corresponding author on reasonable request.
